# High‐yield upcycling of feather wastes into solid‐state ultra‐long phosphorescence carbon dots for advanced anticounterfeiting and information encryption

**DOI:** 10.1002/EXP.20230166

**Published:** 2024-05-14

**Authors:** Dongzhi Chen, Xin Guo, Xuening Sun, Xiang Feng, Kailong Chen, Jinfeng Zhang, Zece Zhu, Xiaofang Zhang, Xin Liu, Min Liu, Li Li, Weilin Xu

**Affiliations:** ^1^ State Key Laboratory of New Textile Materials and Advanced Processing Technology Wuhan Textile University Wuhan Hubei P. R. China; ^2^ School of Materials Science and Engineering Wuhan Textile University Wuhan Hubei P. R. China; ^3^ Institute of Super‐Microstructure and Ultrafast Process in Advanced Materials School of Physics and Electronics Central South University Changsha Hunan P. R. China; ^4^ School of Textiles and Clothing The Hong Kong Polytechnic University Hong Kong P. R. China

**Keywords:** anti‐counterfeiting ink, carbon dots, feather wastes, room‐temperature phosphorescence, solid‐state fluorescence

## Abstract

Recently, biomass‐derived carbon dots (CDs) have attracted considerable attention in high‐technology fields due to their prominent merits, including brilliant luminescence, superior biocompatibility, and low toxicity. However, most of the biomass‐derived CDs only show bright fluorescence in diluted solution because of aggregation‐induced quenching effect, hence cannot exhibit solid‐state long‐lived room‐temperature phosphorescence (RTP) in ambient conditions. Herein, matrix‐free solid‐state RTP with an average lifetime of 0.50 s is realized in the CDs synthesized by one‐pot hydrothermal treatment of duck feather waste powder. To further enhance RTP lifetime, hydrogen bonding is introduced by employing polyols like polyvinyl alcohol (PVA) and phytic acid (PA), and a bimodal luminescent CDs/PVA/PA ink is exploited by mixing the CDs and polyols. Astonishingly, the CDs/PVA/PA ink screen‐printed onto cellulosic substrates exhibits unprecedented green RTP with average lifetime of up to 1.97 s, and the afterglow lasts for more than 14 s after removing UV lamp. Such improvement on RTP is proposed to the populated excited triplet excitons stabilized by rigid chains. Furthermore, the CDs/PVA/PA ink demonstrates excellent potential in anticounterfeiting and information encryption. To the best of the authors' knowledge, this work is the first successful attempt to fabricate matrix‐free ultra‐long RTP CDs by reclamation of the feather wastes for environmental sustainability.

## Graphical abstract

Matrix‐free solid‐state RTP with an average lifetime of 0.50 s is realized in the carbon dots (CDs) synthesized by the one‐pot hydrothermal process of powder made of feather wastes. The room‐temperature phosphorescence lifetime is further prolonged to 1.97 s by employing polyvinyl alcohol (PVA) and phytic acid (PA). Subsequently, a novel CDs/PVA/PA ink is successfully exploited and applied in anticounterfeiting and information encryption.

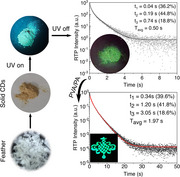

## 1 INTRODUCTION

Globally, more than 8.6 million tons of keratinous waste, mainly poultry feathers and animal hairs, are produced annually from the meat industry.^[^ ^1^ ^]^ Particularly, the increasing demand for poultry meat generates massive amounts of feather wastes,^[^ ^2^ ^]^ causing huge pressure on the environment. Due to strong disulfide bridges, hydrogen bonds, and hydrophobic interactions between polypeptide chains, the discarded feathers are non‐degradable, leading to a formidable challenge for disposing of the feather waste. Until now, there have been two ways to dispose feather wastes. The first way is to landfill or incinerate the feather waste, which could be an efficient strategy for treating the discarded feathers quickly. But landfilling or incineration not only wastes lots of invaluable biomass resources, but also causes environmental pollution like gas releases (such as ammonia and greenhouse gas)^[^ ^2a^ ^]^ The other pathway is to upcycle feather wastes into useful products, which are becoming an attractive focus for achieving sustainable development. To date, the wasted feathers have been applied to diverse fields, including feedstuff, construction, fertilizers, and so forth.^[^ ^2^, ^3^ ^]^ But these applications are mainly focused on low‐value products, and seldom involve high‐end demands. Therefore, it is highly desirable to recycle feather wastes into high added‐value functional materials. Recently, goose feathers have been reported to be fabricated carbon‐based fluorescent probes for the detection of Fe^3+^ ions, providing tutorial information on exploring luminescent materials.^[^ ^4^ ^]^

In recent years, carbon dots (CDs) have attracted tremendous attention in numerous fields such as anti‐counterfeiting, bioimaging, light‐emitting diodes, and so forth, due to their prominent merits, including brilliant luminescence, superior biocompatibility, and low toxicity.^[^ ^5^ ^]^ However, most of the CDs usually suffer from aggregation‐induced‐quenching effect, and seldom exhibit long‐lived room‐temperature phosphorescence (RTP) in a solid state. Moreover, syntheses of the CDs usually undergo time‐consuming procedures, severely hindering real‐life applications.^[^ ^6^ ^]^ If these issues are properly addressed, the CDs will completely work as desirable multimodal luminescent platforms for future advanced applications.

Generally, there are two methods to improve luminescence duration. One is to promote intersystem crossing (ISC) transitions of the excitons from the excited singlet to triplet states by enhancing the spin−orbit coupling (SOC), which is usually realized by doping some heavy atoms (e.g., Cl, Br, or I) or heteroatoms (e.g., N, O, S, or P) into CDs.^[^ ^7^ ^]^ The other is to suppress nonradiative transitions (molecular rotations or vibrations) of the excitons from the excited triplet states to the ground state,^[^ ^8^ ^]^ or to prevent oxygen quenching. To date, varieties of interactions, including hydrogen bonding, ionic bonding, covalent bonding and so forth, have been extensively utilized for creating rigid environments to suppress the nonradiative decays of CDs.^[^ ^6^, ^9^ ^]^ To achieve the confinement effect, the CDs are commonly incorporated into some multiple H‐bonding matrices, such as poly (vinyl alcohol),^[^ ^6^, ^10^ ^]^, nano‐silica^[^ ^11^ ^]^, zeolites^[^ ^12^ ^]^, cyanuric acid^[^ ^13^ ^]^, etc. But these CDs composites only show a long afterglow with the help of the rigid matrices. Fortunately, the CDs simultaneously with solid‐state fluorescence and RTP have been reported, but the RTP life‐time only spans several milliseconds.^[^ ^14^ ^]^ Therefore, it is still a formidable challenge to explore matrix‐free solid‐state long‐lived RTP carbon‐based materials.

To address the forementioned issues, novel biomass‐derived CDs (high yield: 89.6 %) possessing simultaneously matrix‐free solid‐state fluorescence and RTP are exploited by one‐step hydrothermal treatment of the discarded duck feather powder. The resultant CDs not only exhibit impressive blue fluorescence in aqueous‐dispersion under 365 nm UV irradiation, but also emit fascinating matrix‐free solid‐state green RTP with an average lifetime of 0.50 s after turning off the UV lamp. To further enhance RTP life‐time, hydrogen bonding is introduced by employing polyols, including polyvinyl alcohol (PVA) and phytic acid (PA). Subsequently, novel CDs/PVA/PA inks are fabricated by blending PVA/PA and the CDs, and their printability is optimized by rheological properties. To examine their practicability for security applications, the customized patterns are screen‐printed onto typical cellulosic substrates, including cotton fabric and cellulosic paper using the CDs/PVA/PA ink. Astonishingly, the CDs/PVA/PA ink printed on cotton fabric exhibits unprecedented green RTP with an average lifetime of 1.97 s, and an afterglow duration of up to about 14 s. Notably, the CDs/PVA/PA ink can fully hide the customized patterns and encoding information, demonstrating huge potential in security fields. As a paramount work, these findings not only afford a large‐scalable method to synthesize the CDs possessing simultaneously matrix‐free solid‐state fluorescence and RTP, but also contribute to the sustainability for the reuse of the discarded feathers.

## 2 EXPERIMENTAL SECTION

### 2.1 Materials

Superfine duck feather powder (particle size: 2.53 µm) was obtained by smashing the discarded feathers in our laboratory. Both polyvinyl alcohol (PVA) (polymerization degree, 1750 ± 50) and phytic acid (PA) (70% water solution) were purchased from Sinopharm Chemical Reagent Co., Ltd. Absolute ethanol (AR) was purchased from Shanghai Sinopharm Chemical Reagent Co., Lit. Deionized water (18.2 MΩ cm) was used throughout the experiments. All reagents were used as received without further purification.

### 2.2 Synthesis of the feather‐derived CDs

0.4 g duck feather powder was dispersed into 50 mL of deionized water in a 100 mL beaker with stirring for 12 h. Afterward, the mixture was transferred into a stainless‐steel autoclave with Teflon‐lined liner (100 mL capacity), and heated at 220°C for 10 h. Subsequently, the stainless‐steel autoclave was cooled to room temperature in ambient conditions. The light‐yellow liquid was obtained through microporous membrane (0.05 µm) filtration. The concentrated filtrate was purified via silica column chromatography using a mixture of deionized water and ethanol as the eluent (the volume ratio is equal to 1:1). Finally, the collected eluent was concentrated and freeze‐dried to afford the feather‐derived solid CDs with yield of up to 89.6%.

### 2.3 Fabrication of the CPP inks

Firstly, PVA (5.0 g) and deionized water with variable mass were charged into a round‐bottom flask equipped with a mechanical stirrer, respectively. The mixture was mechanically stirred for 2 h at 60°C to afford PVA solution. Then, 3.0 g PA was added into the PVA solution with mechanical stirring for 1.5 h to afford PVA/PA solution. Finally, 5.0 g dispersion of CDs (3%) was added into the PVA/PA solution, and continuously stirred to formulate homogenous CDs/PVA/PA (CPP) inks. For comparison, CDs/PVA (CP‐0) ink without adding PA was also fabricated under the same conditions. The detailed recipes of the CPP inks are listed in Table 1.

**TABLE 1:** The formulations of the CPP inks.

Sample	PVA (g)	Water (g)	PA (g)	Dispersion of CDs (g)	Weight percentage of PVA (%)	Weight percentage of CDs (%)
CPP‐1	5.0	58.4	3.0	5.0	7%	0.21
CPP‐2	5.0	37.0	3.0	5.0	10%	0.30
CP‐0	5.0	40.0	/	5.0	10%	0.30
CPP‐3	5.0	28.7	3.0	5.0	12%	0.36
CPP‐4	5.0	22.7	3.0	5.0	14%	0.42

Abbreviations: CDs, carbon dots; CP, CDs/PVA; CPP, CDs/PVA/PA; PA, phytic acid; PVA, polyvinyl alcohol.

### 2.4 Preparation of the labels with the customized information

To check its practicability for security applications, customized information with diverse patterns such as orchid grass, bamboo, solid‐circle, and campus QR code was screen‐printed on cotton fabric and cellulosic paper using CPP‐2 ink, respectively. After printing, all these labels were obtained by drying in ambient conditions for 30 min.

### 2.5 Characterization

Morphologies of the duck feather‐derived CDs were characterized using a JEOL‐2100 transmission electron microscopy (TEM) (JEOL, Japan) under 200 kV. The CDs were ultrasonically dispersed in deionized water for 15 min, and the supernatant of the CDs was deposited on a copper grid coated with carbon film. Fourier transform infrared (FTIR) spectra of the CDs and duck feather powder were recorded using the FTIR spectrometer (Nicolet is50, USA) in the range of 4000 to 400 cm^−1^ in attenuated total reflectance mode at room temperature. The Raman spectrum for the CDs was obtained on a LabRAM HR Evolution Raman spectrometer (HORIBA Scientific, Japan). Ultraviolet/visible (UV/vis) absorption spectra of the CDs dispersed in water and the aggregated solid‐state CDs were recorded using UV 1000F (Labsphere, USA), respectively. X‐ray photoelectron spectra (XPS) of the CDs using a Thermo Scientific (K‐Alpha, USA) scanning X‐ray photoelectron spectrometer microprobe. Powder X‐ray diffraction (XRD) pattern of the CD was recorded on an X‐ray diffractometer (Holland Empyrean) with Cu Ka radiation. Room‐temperature fluorescent and phosphorescent spectra of the specimens were recorded by a Hitachi fluorescence spectrophotometer (F‐4700). The phosphorescence spectra at varying temperatures were measured using FLS1000 (Edinburgh, UK) spectrofluorometers equipped with a microsecond flash‐lamp (µF900), the sample was placed in a low‐temperature attachment (Optistat DN, Oxford) and the temperature was controlled between 300 and 77 K. The spectral characterization of absolute PL quantum yield (QY) was measured using a custom‐designed integrating sphere (Jinan Gen Spectrum Instrument Co., Ltd) with an inner diameter of 50 mm.^[^ ^15^ ^]^ The CDs aqueous dispersion or powder was filled into a quartz tube inserted in the integrating sphere, excited by a UV LED (365 nm, Shen Zhen UVSIS Technology Co., Ltd) with a narrow band filter (BP365‐10 nm, Rayan Technology Co., Ltd). A spectrometer (NOVA, Ideaoptics, Inc.) was used for measuring the luminescence spectra, and the wavelength‐dependent spectral responsivity of the system was calibrated by a tungsten halogen lamp. During the rubbing experiment, abrasive paper (1200 mesh) was used as an abrasive surface, the quick response (QR) code printed on cellulosic substrates was placed faced‐down onto abrasive paper under loading of 500 g, and the sample was dragged back and forth in a straight line along with a 10.4 cm track. One back and forth defines a time.^[^ ^16^ ^]^

The absolute PL QY was calculated according to Equation (1):^[^ ^5j^ ^]^

(1) $$\mathrm{QY}=\frac{N_{Em}}{N_{Abs}}=\frac{P_{s}-P_{b}}{L_{b}-L_{s}}$$

Where $N_{Abs}\;$ is the number of photons absorbed by the sample, $N_{Em}$ is the number of photons emitted from the sample. *P_s_* and *P_b_* are the integral area of the emission band of the sample and the blank sample, respectively. *P_s_*–*P_b_* is the integral area of the emission photon profile. *L_s_* and *L_b_* are the integral area of the excitation band of the sample and the blank sample, respectively. *L_b_*−*L_s_* is the integral area of the excitation photon profile.

The molecular orbital energy gap ($\Delta E_{\mathrm{st}})$ of the water‐dispersed CDs and aggregated solid‐state CDs are calculated according to Equation (2) based on their fluorescence (FL) and phosphorescence (Phos) emission spectra ^[^ ^13^, ^14^ ^]^:

(2) $$\Delta \;E_{\mathrm{st}}=\frac{1240}{\lambda_{FL}}\;-\frac{1240}{\lambda_{Phos}}$$

Where $\Delta E_{\mathrm{st}}$ is the energy gap between the lowest singlet and triplet exited states. $\lambda_{Phos}$ and $\lambda_{FL}$ are the wavelength maxima of phosphorescence and fluorescence emissions, respectively.

## 3 RESULTS AND DISCUSSION

### 3.1 Fabrication, morphology, and composition of the feather‐derived CDs

The concentrated aqueous‐dispersion of the CDs is clear, transparent, pale‐yellow under daylight, while it emits deep blue fluorescence with a QY of ≈17% under 365 nm UV illumination. After freeze‐drying, the dried CDs are in the form of brown powder under daylight, which can emit malachite‐green fluorescence with a QY of ≈12% under 365 nm UV irradiation. Fascinatingly, it can exhibit bright‐green RTP after UV irradiation, as shown in Figure S1.

**FIGURE 1:**
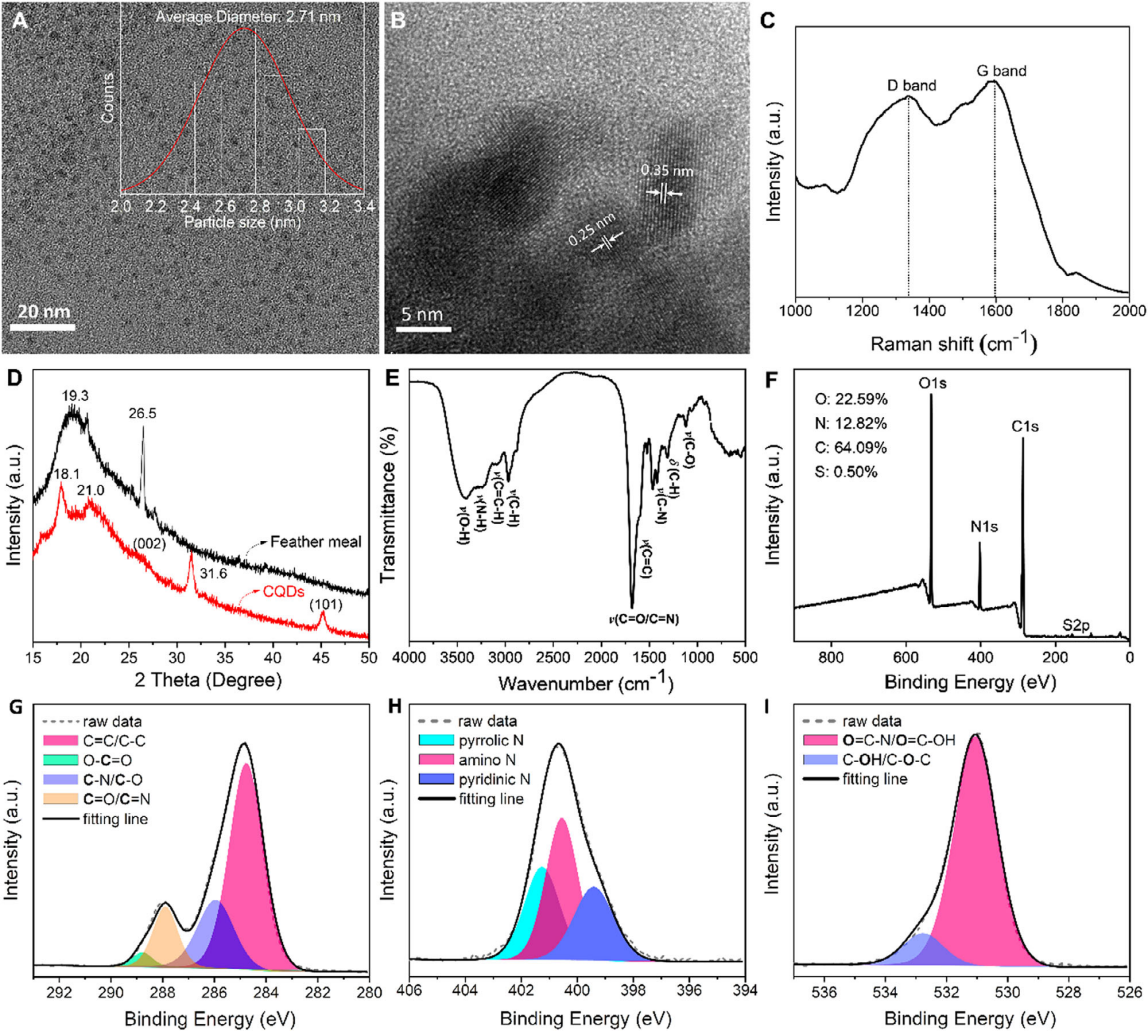
(A) TEM image of the resultant carbon dots (CDs), and the inset is the statistical size‐distribution of the CDs, (B) high‐resolution TEM image of the CDs. (C) Raman spectrum of the solid‐state CDs. (D) XRD pattern of duck feather powder and the CDs powder, (E) FTIR spectrum, and (F) full XPS survey spectrum of CDs. High‐resolution XPS spectra and fitting results of the CDs: C 1s (G), N 1s (H), and O 1s (I).

Figure 1A clearly exhibits that the resultant CDs present quasi‐spherical nanoparticles with an average size of 2.71 nm. A high‐resolution TEM image clearly reveals that the CDs have two types of lattice fringe spacings of 0.25 and 0.35 nm, which are attributable to the (111) and (002) planes of graphite,^[^ ^17^ ^]^ respectively, as shown in Figure 1B. These characters verify that the resultant CDs have graphite‐like structures. Subsequently, the Raman spectrum of the CDs exhibits two characteristic bands at 1590 and 1340 cm^−1^, attributable to the *E*_2g_ vibrational modes of the sp^2^ hybridized carbon domains (G band), and the breathing vibrations of carbon atoms with dangling bonds in the termination plane of disordered graphite domains (D band),^[^ ^18^ ^]^ respectively. The intensity ratio of the D band to the G band (*I*_D_/*I*_G_) is 0.79, suggesting that the resultant CDs are composed of high‐quality crystalline graphitic cores and amorphous graphitic domains, as shown in Figure 1C. Further, powder XRD was also employed to characterize the CDs. As shown in Figure 1D, the XRD pattern of duck feather powder shows a broad peak at around 19.3° and a sharp peak at 26.5° referring to β‐sheet of keratins.^[^ ^3^, ^19^ ^]^ After hydrothermal reaction, some new diffraction peaks at 18.1°, 21.0°, and 31.6° are observed, respectively. These diffraction patterns are relative to features of amorphous graphitic carbon, which might result from small molecules formed during the pyrolysis and carbonization of keratins upon hydrothermal process.^[^ ^20^ ^]^ Meanwhile, the typical diffraction peaks at around 26.6° and 45.3° attributable to the (002) and (101) lattice planes of graphitic carbon are observed,^[^ ^21^ ^]^ respectively. Those results consistently confirm that the feather‐derived CDs are mostly composed of crystalline and amorphous graphitic carbons.

To gain insight into surface groups, the feather‐derived CDs were systemically characterized by FTIR and XPS, respectively. As shown in Figure 1E, the FTIR spectrum shows two broad absorption bands at around 3400 and 3200 cm^−1^ attributed to the stretching vibrations of hydrogen‐bonded O─H and N─H groups, respectively.^[^ ^17e^ ^]^ The bands at 3080, 2960, and 2876 cm^−1^ are assigned to the stretching vibrations of the unsaturated C─H and saturated C─H groups,^[^ ^5c^ ^]^ respectively. The absorption bands at around 1596, 1518, and 1455 cm^−1^ are ascribed to the typical skeleton vibrations of the conjugated aromatic rings, indicating carbonization occurred during the hydrothermal process. The absorption bands located at around 1667, 1411, and 1117 cm^−1^ attributable to the stretching vibrations of C═O/C═N, C─N and C─O, respectively,^[^ ^10^, ^14^, ^22^ ^]^ indicate that the resultant CDs are abundant in nitrogenous and oxygen‐containing groups. Subsequently, the full survey of the XPS spectrum of the CDs shows four elements such as S (0.50%), C (64.09%), N (12.82%), and O (22.59%) at 163.4, 284.8, 400.64, and 530.78 eV, respectively, confirming that the CDs are composed of C, O, N, and S, as shown in Figure 1F. It is noteworthy that the oxygen and nitrogen atomic percentages increase after the hydrothermal process compared to duck feather powder in Figure S2, suggesting that hydrolytic condensation and oxidation reactions occurred. Meanwhile, the carbon and sulfur amounts of the CDs are dropped, implying that cleavage and oxidation reactions of keratin also occurred. Subsequently, Figure 1G shows that the high‐resolution spectrum in the C1s band is deconvoluted into four peaks at 284.8, 285.9, 288.0, and 288.8 eV, which are assigned to C─C/C═C, **C**─N/**C**─O/**C**─S, **C**═N/**C**═O, and O═**C**─OH, respectively.^[^ ^5^, ^23^ ^]^ Afterward, the high‐resolution N 1s spectrum reveals three peaks at 399.4, 400.5, and 401.2 eV attributable to pyridinic N, amino N, and pyrrolic N, respectively,^[^ ^22^ ^]^ as shown in Figure 1H. Accordingly, the O 1s spectrum shows two peaks at 531.6 and 532.8 eV (Figure 1I), which are ascribed to **O**═C─N/**O**═C─OH, and C─**O**H,^[^ ^5a^ ^]^ respectively. Additionally, the S 2p spectrum can be fitted into two peaks, corresponding to C─**S**─C (164.0 eV) and ─C─**S**O*_x_* ─ (*x* = 2, 167.1 eV; *x* = 3, 168.1 eV, *x* = 4, 169.1 eV) species,^[^ ^4^, ^24^ ^]^ respectively, as shown in Figure S3. Collectively, profuse nitrogenous and oxygen‐containing groups are on the surface of the CDs.

### 3.2 Optical properties of the resultant CDs

**FIGURE 2:**
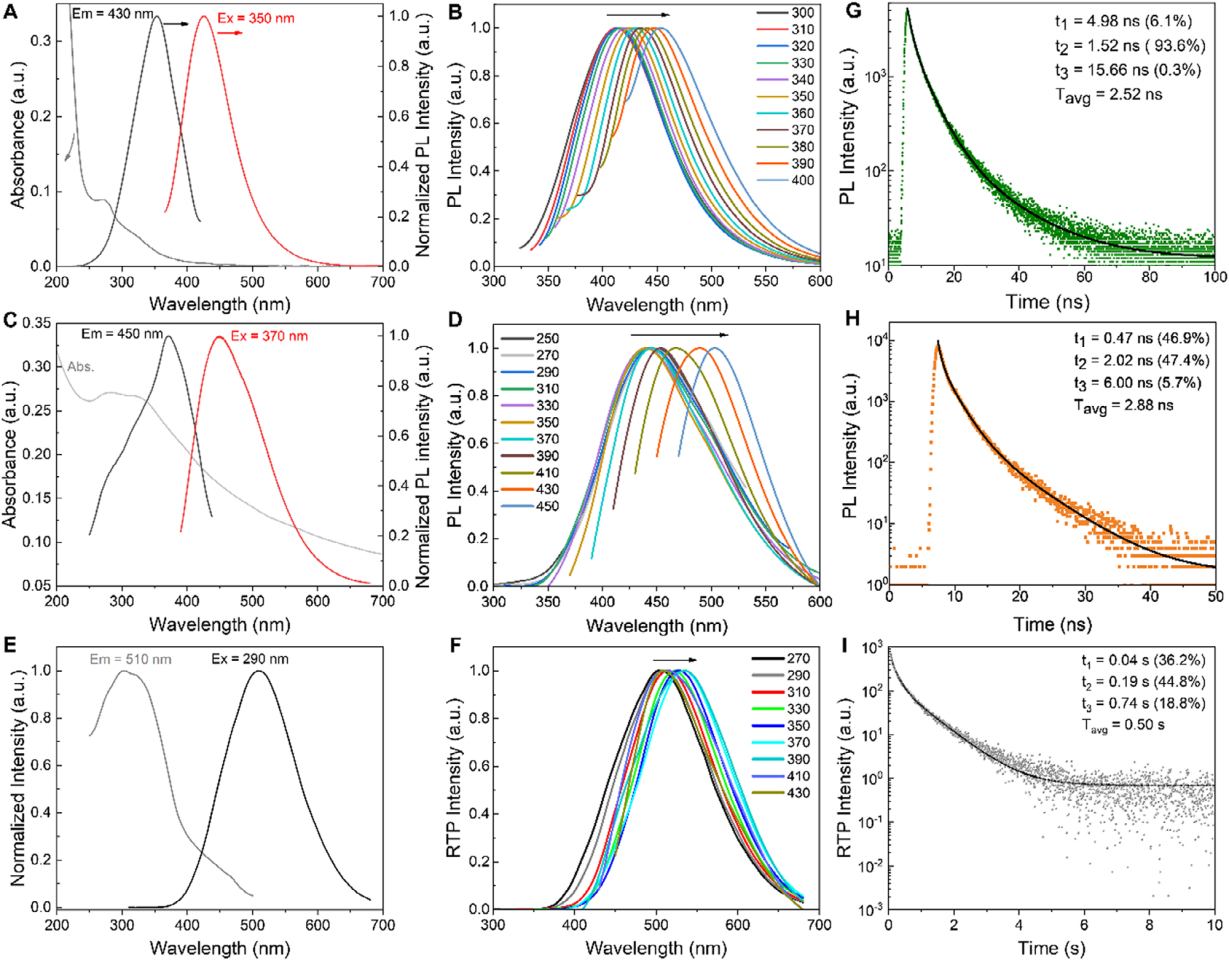
(A) UV/vis absorption, fluorescence excitation and PL emission spectra of the CDs dispersed in water, (B) PL emission spectra of the CDs dispersed in water under different excitation wavelengths, (C) UV/vis absorption, fluorescence excitation and PL emission spectra of the solid‐state powder CDs, (D) PL emission spectra of the solid‐state CDs under different excitation wavelengths, (E) RTP excitation and emission spectra of the solid‐state CDs, (F) RTP emission spectra of the solid‐state CDs under different excitation wavelengths. PL decay spectra of the CDs dispersed in water (G), recorded at emission wavelength of 430 nm under excitation at 350 nm, and the solid‐state CDs (H), recorded at emission wavelengths of 450 nm under excitation at 370 nm. RTP decay spectrum of the solid‐state CDs (I), recorded at emission wavelengths of 510 nm under excitation at 290 nm. Em, emission wavelength; Ex, excitation wavelength; T_avg_, average lifetime.

The UV/vis absorption spectra of the water‐dispersed CDs and the solid‐state CDs obviously exhibit two similar broad absorption bands at around 280 and 320 nm, which are attributed to the π–π* transitions of the conjugated C═C and C═N bonds in the graphitized carbon cores,^[^ ^14^, ^23^, ^25^ ^]^ and the n‐π* transitions of the sub‐fluorophores (e.g., ─NH_2_, ─C─OH, ─C═O and ─C═N) on surfaces of the CDs,^[^ ^17^, ^26^ ^]^ respectively, as shown in Figure 2A,C. The PL spectrum of the water‐dispersed CDs exhibits the emission maximum centered at 430 nm under excitation wavelength of 350 nm. It is easily found that both the water‐dispersed and the solid‐state CDs show analogous excited‐dependent optical properties, implying that the same factors (core state and surface state) contribute to the PL origin of both the water‐dispersed and the solid‐state CDs, as shown in Figure 2B,D and Figure S4A,B,E. Interestingly, the absorption bands become broad, and bathochromic phenomena (20 nm) are respectively observed in the absorption and PL spectra of the CDs when transferred from the aqueous‐dispersion to the aggregated solid‐state, indicating that interparticle distance becomes short and new multiple energy levels probably form due to hydrogen bonding.

Subsequently, the RTP spectra of the aggregated solid‐state CDs strikingly exhibit an emission maximum centered at around 510 nm under an excitation wavelength of 290 nm, as shown in Figure 2E. The RTP excitation spectrum of the solid‐state CDs covers the whole absorption band ranging from 250 to 500 nm (Figure 2C), confirming that the RTP mainly originates from the π–π* transitions of the graphited cores and the n‐π* transitions of the surface sub‐fluorophores from the CDs. Meanwhile, the maximum emission peak for the solid‐state CDs also shows redshifts with excitation wavelength, indicating excited‐dependent RTP for the solid‐state CDs, as shown in Figure 2F and Figure S4C,F. The redshifts in both photoluminescence and RTP may be due to the increasing amount of oxygen species on the surfaces of the CDs,^[^ ^23a^ ^]^ as evidenced in Figure 2F and Figure S2.

The fluorescence and phosphorescence decay spectra of the water‐dispersed CDs and solid‐state CDs are present in Figure 2G–I, respectively. All fluorescence and phosphorescence decay curves are fitted as a similar triple exponential function according to Equation (3), respectively.^[^ ^6c^ ^]^ Meanwhile, average lifetimes were also calculated by Equation (4).^[^ ^13^ ^]^ Interestingly, an average lifetime for fluorescence of the water‐dispersed CDs is calculated as 2.52 ns, while it increases to 2.88 ns when the CDs are in the form of solid powder, as shown in Figure 2G,H. The surfaces of the solid CDs retained plentiful nitrogenous and oxygen‐containing groups, which could control inter/intra‐particle distance by hydrogen bonding, therefore weakening the direct π–π interaction and non‐radiative cross relaxation between the CDs, thus achieving self‐quenching‐resistant solid‐state PL characteristics.^[^ ^27^ ^]^ This results in a much longer lifetime compared to the aqueous dispersion of CDs. Notably, the RTP average lifetime of the solid‐state CDs is up to 0.50 s, outperforming that of most biomass‐derived carbon dots, as shown in Figure 2I and Table S1. Therefore, the bimodal luminescence features for the CDs provide great potential in future advanced applications.

(3) $$\Upsilon =\Upsilon_{0}+A_{1}*e^{\frac{-\left(x-x_{0}\right)}{t_{1}}}+A_{2}*e^{\frac{-\left(x-x_{0}\right)}{t_{2}}}+A_{3}\;*e^{\frac{-\left(x-x_{0}\right)}{t_{3}}}$$

(4) $$T_{\mathrm{average}}=\frac{A_{1}t_{1}^{2}+A_{2}t_{2}^{2}+A_{3}t_{3}^{2}}{A_{1}t_{1}+A_{2}t_{2}+A_{3}t_{3}}$$

### 3.3 Rheological properties and anti‐counterfeiting applications of CPP inks

**FIGURE 3:**
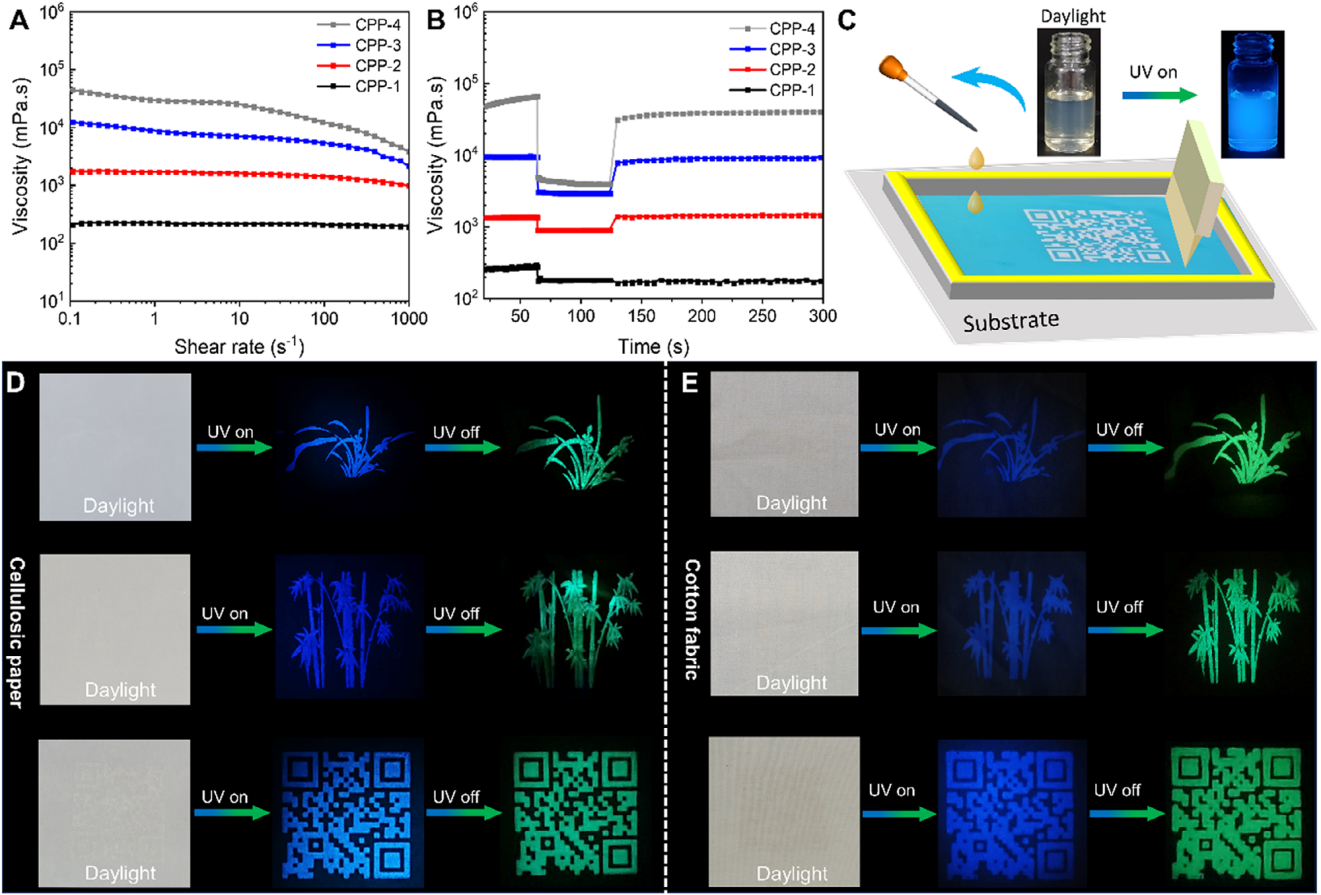
Rheological properties of the CPP inks with variable weight percentages of the resultant CDs: (A) the relationship between viscosity and shear rate, (B) shear viscosity versus time at three different shear rates (0.1 s^−1^ for 60 s, 1000 s^−1^ for 60 s, and 0.1 s^−1^ for 10 min). (C) Photographic illustrations for the screen‐printing process. The photos of the various customized patterns including orchid grass, bamboo and campus QR code transferred on cellulosic paper (D), and cotton fabric (E) by using screen‐printing techniques under daylight, 365 nm UV lamp on, and off. CPP, CDs/PVA/PA; UV, ultraviolet.

It is well‐known that the rheological properties of the ink play a vital role in the screen‐printing process. Figure 3A shows that all the CPP inks exhibit an increasing trend in viscosity at the same shear rate with an increase in the mass percentage of PVA. Meanwhile, viscosities of the CPP inks decrease with shear rate when the PVA percentage exceeds 10%, implicating shear‐thinning thixotropic behavior.^[^ ^16^, ^28^ ^]^ The decreasing viscosity should be ascribed to the dissociated hydrogen bonding among PVA, CDs, and PA under high‐shear force, which facilitates the screen‐printing process. Interestingly, under the same percentage of the CDs, the viscosity of the CP‐0 is much lower than that of the CPP‐2 at the same shear rate, which corroborates the existence of multiple hydrogen‐bonding interactions among PVA, CDs, and PA, as shown in Figure S5. Subsequently, the thixotropic behavior of the CPP inks is further evaluated by a peak‐hold‐step test consisting of holding the sample at different shear rates for three time intervals, as shown in Figure 3B. Obviously, viscosities of all the CPP inks dropped quickly with the shear rate changing from 0.1 to 1000 s^−1^. As the shear rate was recovered to 0.1 s^−1^, viscosities of all the CPP inks rose due to hydrogen‐bond reconstruction. As listed in Table S2, the recovery ratio for viscosity of the CPP‐2 ink can reach 100% within 10 s after the shear stress was released, indicating that fine patterns can be quickly gained using the CPP‐2 ink in the screen‐printing process. Hence, the CPP‐2 ink is chosen to be used for the screen‐printing process.

As shown in Figure 3C, the CPP‐2 ink is translucent under daylight, while it emits blue fluorescence under UV lamp illumination. Subsequently, to examine the practicability of the CPP‐2 ink, various patterns, including orchid grass, bamboo, and campus QR codes are screen‐printed onto surfaces of cellulosic substrates (cotton fabric and cellulosic paper), respectively, as shown in Figure 3D,E. It is noteworthy that no striking pattern is visible on surfaces of cellulosic substrates under daylight, while deep‐blue fluorescent patterns can be clearly discernible on cellulosic substrates under a 365 nm UV lamp. Interestingly, these patterns printed on cellulosic substrates can emit impressively green RTP after ceasing UV irradiation. Especially the fine luminescent patterns, including bamboo leaves and orchid grass, are clearly observable, exhibiting that the CPP‐2 ink possesses excellent practicability on cellulosic substrates.

To examine the fastness of the CPP‐2 ink, the PL intensity of the selected QR code pattern screen‐printed on the representative substrates (cotton textile and cellulosic paper) was evaluated by a rubbing test. As shown in Figure S6, the PL intensity of the printed pattern on the cellulosic paper reduces to 0.81 ± 0.06 after rubbing with abrasive paper for 100 times, exhibiting that CPP‐2 ink printed on cellulosic paper has excellent fastness. Similarly, the PL intensity of the printed pattern on the wetted and dry cotton fabric decreases to 0.61 ± 0.04 and 0.66 ± 0.06 after rubbing with abrasive paper for 100 times, respectively, proving that the CPP‐2 ink has excellent dry and wet fastness. However, the PL intensity of CPP‐2 ink printed on cotton fabric decreases, much lower than that on cellulose paper substrates, due to the rough surface of the cotton fabric, which caused some damage to the surface CPP‐2 ink layer on the cotton substrate by the abrasive paper. Collectively, the excellent fastness further demonstrates that the CPP‐2 ink has universal practicability.

Therefore, these bimodal luminescent features impart the CPP‐2 ink with excellent concealment performance during real‐life application, envisioning that it will be applied to many advanced fields such as valuable documents, luxury items, fingerprint identifications, and so on.

### 3.4 The luminescence mechanisms

**FIGURE 4:**
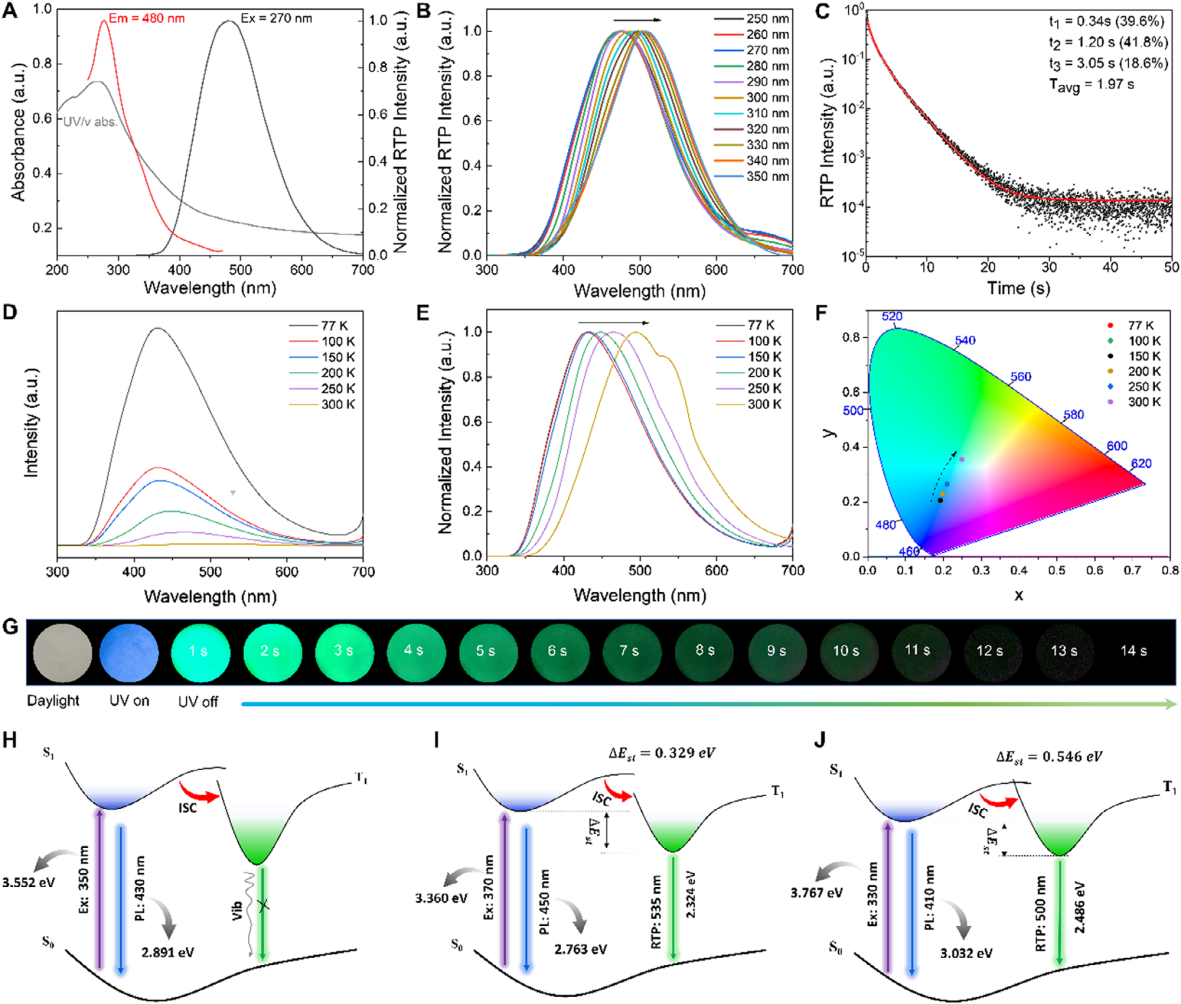
(A) UV/vis absorption, RTP excitation and emission spectra of the customized pattern screen‐printed with CPP‐2 ink on cotton fabric, (B) RTP emission spectra of the customized pattern screen‐printed on cotton fabric with CPP‐2 ink under variable excitation wavelengths, and (C) RTP decay spectrum of the CPP‐2 ink printed on cotton fabric, recorded at emission wavelength of 470 nm under excitation at 270 nm. (D) Phosphorescence spectra of the pattern screen‐printed with CPP‐2 ink on cotton fabric at different temperatures under 270 nm excitation. (E) Normalized phosphorescence spectra of the pattern screen‐printed with CPP‐2 ink on cotton fabric at different temperatures. (F) CIE 1931 chromaticity diagram of the pattern screen‐printed with CPP‐2 ink on cotton fabric at different temperatures. (G) The photos of the printed solid‐circle on cellulosic paper using CPP‐2 ink under daylight, 365 nm UV lamp on, and off. Diagram for the luminescence mechanisms of the feather‐derived CDs dispersed in water (H), aggregated solid‐state CDs (I), and the CPP‐2 ink on cotton fabric (J). Em, emission wavelength; Ex, excitation wavelength; ISC, intersystem crossing; PL, photoluminescence; RTP, room temperature phosphorescence; T_avg_, average lifetime.

The UV/vis absorption spectrum of the customized pattern printed on the cotton fabric using CPP‐2 ink exhibits two discernible broad absorption bands at around 225 and 265 nm. Obviously, a pronounced hypsochromic shift in absorption bands (55 nm) is found as compared to that of the aggregated solid‐state CDs, as shown in Figures 4A and 2C. Afterward, the RTP spectra of the customized pattern also show similar blueshifts in both excitation and emission maxima, compared to those of the aggregated solid‐state CDs (Figure 2E). The striking blue‐shift phenomenon should be attributable to the synergistic effect of reconstructed hydrogen bonding among the CDs, phytic acid, PVA and cellulosic substrates, and restriction of the PVA chains. Interestingly, the RTP emission maxima for the customized pattern on cotton fabric also gradually shift from 470 to 510 nm with an increase in excitation wavelength from 250 to 350 nm, demonstrating an excitation‐dependent RTP for CPP‐2 ink, as shown in Figure 4B and Figure S4D. More importantly, the CPP‐2 ink exhibits an unprecedented RTP with an average lifetime of up to 1.97 s, which is far longer than that of the aggregated solid‐state CDs, as displayed in Figures 4C and 2I. Astonishingly, the afterglow of the solid‐circle screen‐printed on cotton fabric can last for about 14 s after turning off the UV lamp, which can be easily captured by the naked eye, as shown in Figure 4G and Video S1.

The temperature‐dependent time‐gated luminescence spectra of the pattern screen‐printed with CPP‐2 ink on cotton fabric are showcased in Figure 4D. It is clearly observed that the phosphorescent emission intensity of the CPP‐2 ink gradually weakens when the temperature increases from 77 to 300 K, confirming that the afterglow originated from phosphorescence rather than thermally activated delayed fluorescence. This can be attributed to the fact that low temperatures weaken the non‐radiative vibrations and populate the triplet excitons.^[^ ^29^ ^]^ Interestingly, the maximum phosphorescence wavelength for the pattern on cotton fabric shows a red shift from 430 to 490 nm with the temperature increased from 77 to 300 K, demonstrating a temperature‐dependent phosphorescence for CPP‐2 ink, as shown in Figure 4E. The CIE 1931 chromaticity diagram (Figure 4F) shows that the color coordinates of the pattern screen‐printed with CPP‐2 ink on cotton fabric are mainly shifted from a blue to a green region with an increase in temperature.

To facilitate explanation of the unique optical features, the luminescence mechanisms of the water‐dispersed CDs, the solid‐state CDs, as well as the CPP‐2 ink, are proposed in Figure 4H–J, respectively. As the CDs are in aqueous‐dispersion form, many dynamic hydrogen bonds between CDs and water are formed, which likely facilitate non‐radiative transitions of sub‐fluorophore groups on the CDs under UV excitation. As a result, no RTP can be observed from the water‐dispersed CDs after removing UV irradiation, as illustrated in Figure 4H. After becoming the aggregated solid‐state, dynamic hydrogen bonding between water and the CDs disappears, and new static hydrogen bonds are easily reconstructed between interparticle and intraparticle by the abundant nitrogen and oxygen‐containing groups on surfaces of the CDs, leading to the formation of compact carbon cores. The reconstructed hydrogen bonding likely facilitates to stabilize the excited triplet species,^[^ ^26a^ ^30^ ^]^ and suppress non‐radiative decay, contributing to RTP, as illustrated in Figure 4I.

Additionally, the excitation energy level of the aggregated CDs (3.360 eV) is lower than that of the water‐dispersed CDs (3.552 eV), which implies that the low multiorbital energy levels were generated in the aggregation of CDs due to hydrogen bonding.^[^ ^14^, ^31^ ^]^ Meanwhile, the energy gap ($\Delta E_{\mathrm{st}}$) of the CDs between the lowest singlet (S_1_) and triplet (T_1_) excited states is calculated, as illustrated in Figure 4I,J. After the CPP‐2 ink was printed on cellulosic substrates, the $\Delta E_{\mathrm{st}}$ of the CDs immobilized on the cellulosic surface was also calculated to be 0.546 eV, which is higher than 0.329 eV for the aggregated‐state CDs. The enlarged $\Delta E_{\mathrm{st}}$ implies that lower multiorbital energy levels were formed, and no reversed ISC process can be found.^[^ ^6^, ^10^, ^12^, ^13^ ^]^ Therefore, the ISC process from the S_1_ to the T_1_ is probably enhanced by the lower multiorbital energy levels due to hydrogen bonding. In addition, the boosting average lifetime for RTP of the CPP‐2 ink demonstrates that the nonradiative transitions of the emissive species are effectively suppressed by multiple hydrogen bonds in the hybrid matrices (PVA/PA/cellulose).

### 3.5 Information encryption and decoding

**FIGURE 5:**
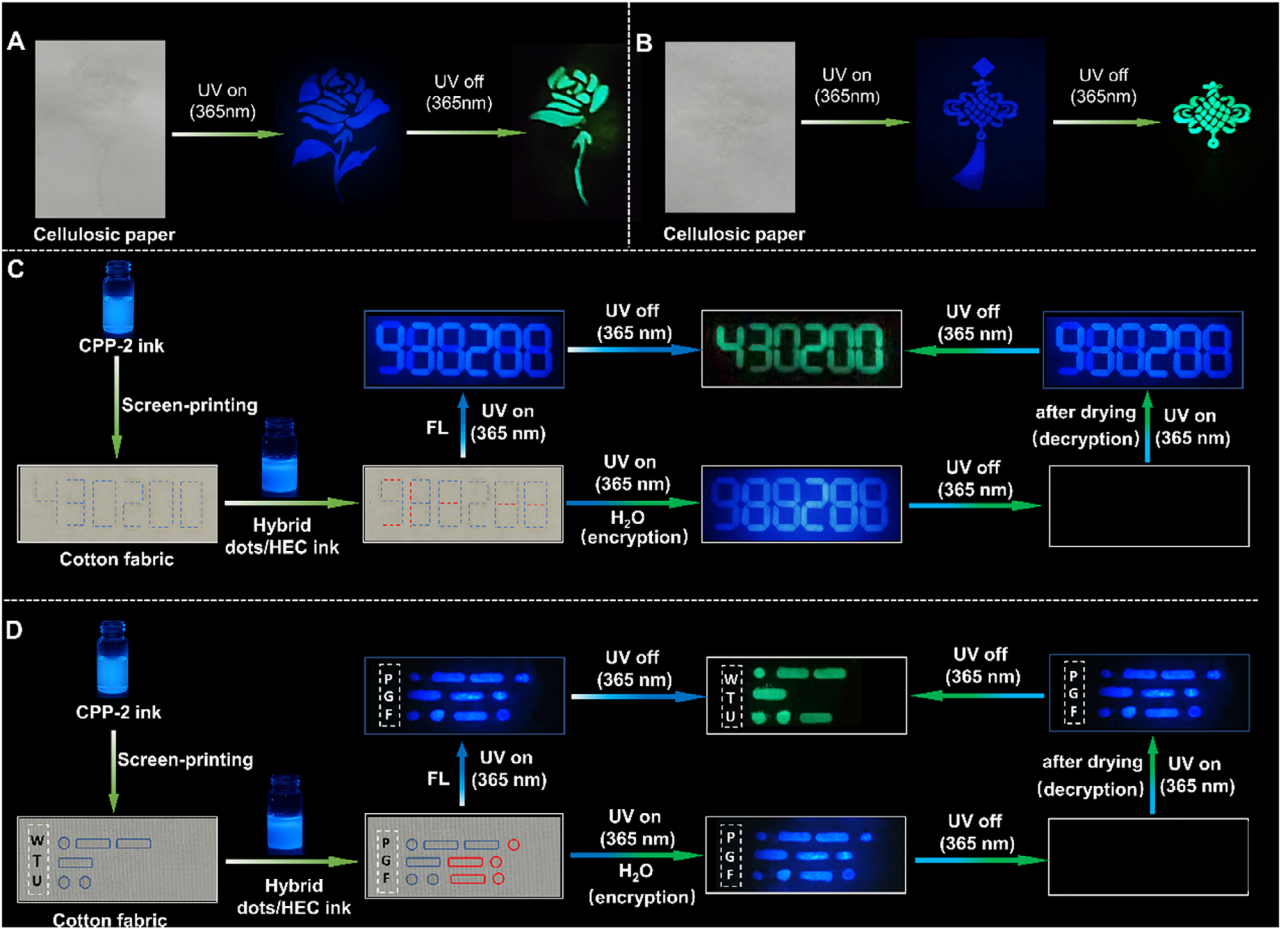
Encryption and decryption of the CPP‐2 ink on cellulosic paper: (A) rose, both the outmost petals and leaves were screen‐printed using the hybrid dots/HEC ink, the other petals and stem were screen‐printed using the CPP‐2 ink, and (B) Chinese knot, its hanger and tassel were screen‐printed using the hybrid dots/HEC ink; only body of knot was screen‐printed using CPP‐2 ink; and cotton fabrics: (C) postal code of affiliation address (e.g., 430200), only the marked red dash lines were screen‐printed using the hybrid dots/HEC ink and (D) Morses codes of WTU, only the red circled domains were filled by screen‐printing technique using the hybrid dots/HEC ink. CPP, CDs/PVA/PA; FL, fluorescence; HEC, hydroxyethyl cellulose; UV, ultraviolet.

By taking advantage of unique afterglow features, the practicability of the CPP‐2 ink in information encryption and decryption is also examined. For comparison, another hybrid dots/hydroxyethyl cellulose (HEC) ink with a similar blue fluorescence is also prepared for interferences, according to the literature.^[^ ^16a^ ^]^ After screen‐printing, a rose with blue fluorescence is distinctly observable on the screen‐printed cellulosic paper under 365 nm UV illumination. Interestingly, after drying, an incomplete rose with a green RTP afterglow is observed after ceasing the UV lamp illumination. Similarly, a complete Chinese knot with blue fluorescence can be screen‐printed on cellulosic paper under 365 nm UV illumination, while only the main part of the knot with green RTP afterglow is discernible after turning off the UV lamp, suggesting that the CPP‐2 ink has excellent encryption, as shown in Figure 5A,B.

To further assess encryption performance, the encrypted postal code of our campus on the fabric label is examined, as shown in Figure 5C. The wrong information on “988288” with blue fluorescence can be read out under 365 nm UV illumination. After turning off the UV lamp, the correct code “430200” with green RTP can be clearly observed. More interestingly, as the encrypted fabric label is wetted by water, the wrong information on “988288” with blue fluorescence is re‐observed under a 365 nm UV lamp, but no information can be found after removing the UV lamp. In this case, the rigidified emissive species are quenched by the reconstruction of dynamic hydrogen bonds among CDs, PVA, cellulose, and water,^[^ ^11a^ ^]^ leading to the RTP disappearance of the CPP‐2 ink. After drying, the wrong information on “988288” on the label reappear under 365 nm UV illumination. While the authentic postal code “430200” with green RTP can be distinctly re‐observable after removing the UV lamp. Figure 5D also presents a more secure multimode encryption according to Morse codes. The encrypted information is invisible under daylight, but it is recognized as “PGF” with blue fluorescence under 365 nm UV illumination. After turning off the UV lamp, the printed pattern exhibits the “WTU” code with green RTP. Similarly, water cannot affect the fluorescent identification of the screen‐printed code on cotton fabric under 365 nm UV lamp, but can completely quench RTP of the CPP‐2 ink after removal of the UV lamp. Only the wet cotton fabric was dried, the hidden authentic code with green RTP can be reidentified after turning off the UV lamp. By contrast, the solid CDs also show a similar water‐quenching RTP phenomenon, as shown in Figure S7. Therefore, the ultralong RTP features can impart the CPP‐2 ink with unlimited possibilities in information encryption and decryption.

## 4 CONCLUSIONS

In summary, novel CDs are fabricated with a yield of up to 89.6% by one‐pot hydrothermal treatment of duck feather waste powder. The resultant CDs not only exhibit aggregated solid‐state fluorescence under UV lamp illumination, but also show matrix‐free green RTP after removing the UV lamp. Subsequently, the chemical compositions, morphology, structure, and optical properties of the resultant CDs are characterized, respectively, finding that the RTP average lifetime of the aggregated solid‐state CDs is up to 0.50 s. To further enhance RTP lifetime, hydrogen bonding is introduced by employing polyols like PVA and PA, and the bimodal luminescent ink was exploited by mixing the feather‐derived CDs and polyols. Afterward, the uses of CDs/PVA/PA ink in anticounterfeiting, information encryption, and decryption are examined on cellulosic substrates (cotton fabric and cellulosic paper), respectively. Astonishingly, the CDs/PVA/PA ink on cotton fabric exhibits an unprecedented RTP lifetime of 1.97 s, and the afterglow can last for about 14 s to the naked eyes after ceasing the UV lamp irradiation. The ultralong RTP features of the CDs/PVA/PA ink are also proposed to populate the excited triplet excitons stabilized by rigid chains due to hydrogen bonding. Notably, CDs/PVA/PA ink can exhibit excellent potential in the fields of advanced anticounterfeiting, information encryption, and decoding. To the best of our knowledge, this work is the first successful attempt to fabricate matrix‐free ultra‐long solid‐state green RTP CDs by reclamation of the feather wastes for environmental protection, envisioning that they will offer unlimited opportunities for future advanced applications in anti‐counterfeiting, information encryption, and so on.

## CONFLICT OF INTEREST STATEMENT

The authors declare no conflicts of interest.

## Supporting information

Supporting Information

Supplemental Video 1

## Data Availability

The data that support the findings of this study are available from the corresponding author upon reasonable request.
