# Supplementary material for: High‐yield upcycling of feather wastes into solid‐state ultra‐long phosphorescence carbon dots for advanced anticounterfeiting and information encryption
Source: Exploration (Beijing). 2024 May 14;4(6):20230166. doi: 10.1002/EXP.20230166 (PMC11655309; doi:10.1002/EXP.20230166)
Supplement: Supplementary file 1 — Supporting Information [file EXP2-4-20230166-s002.docx]

Supporting Information

**High-yield Upcycling of Feather Wastes into Solid-state Ultra-long Phosphorescence Carbon Dots for Advanced Anticounterfeiting and Information Encryption**

Dongzhi Chen*, Xin Guo, Xuening Sun, Xiang Feng, Kailong Chen, Jinfeng Zhang, Zece Zhu, Xiaofang Zhang, Xin Liu, Min Liu, Li Li and Weilin Xu*

D. Chen, X. Guo, X. Sun, J. Zhang, Z. Zhu, X. Zhang, X. Liu, W. Xu

State Key Laboratory of New Textile Materials & Advanced Processing Technology, Wuhan Textile University, Wuhan, 430200, Hubei, P. R. China

E-mail: chdozh_2008@163.com, weilin_xu@wtu.edu.cn

D. Chen, X. Feng, K. Chen, X. Liu

School of Materials Science and Engineering, Wuhan Textile University, Wuhan, 430200, Hubei, P. R. China

M. Liu

Institute of Super-Microstructure and Ultrafast Process in Advanced Materials, School of Physics and Electronics, Central South University, Changsha, 410083, Hunan, P. R. China

L. Li

School of Textiles and Clothing, The Hong Kong Polytechnic University, Hong Kong 999077, P. R. China


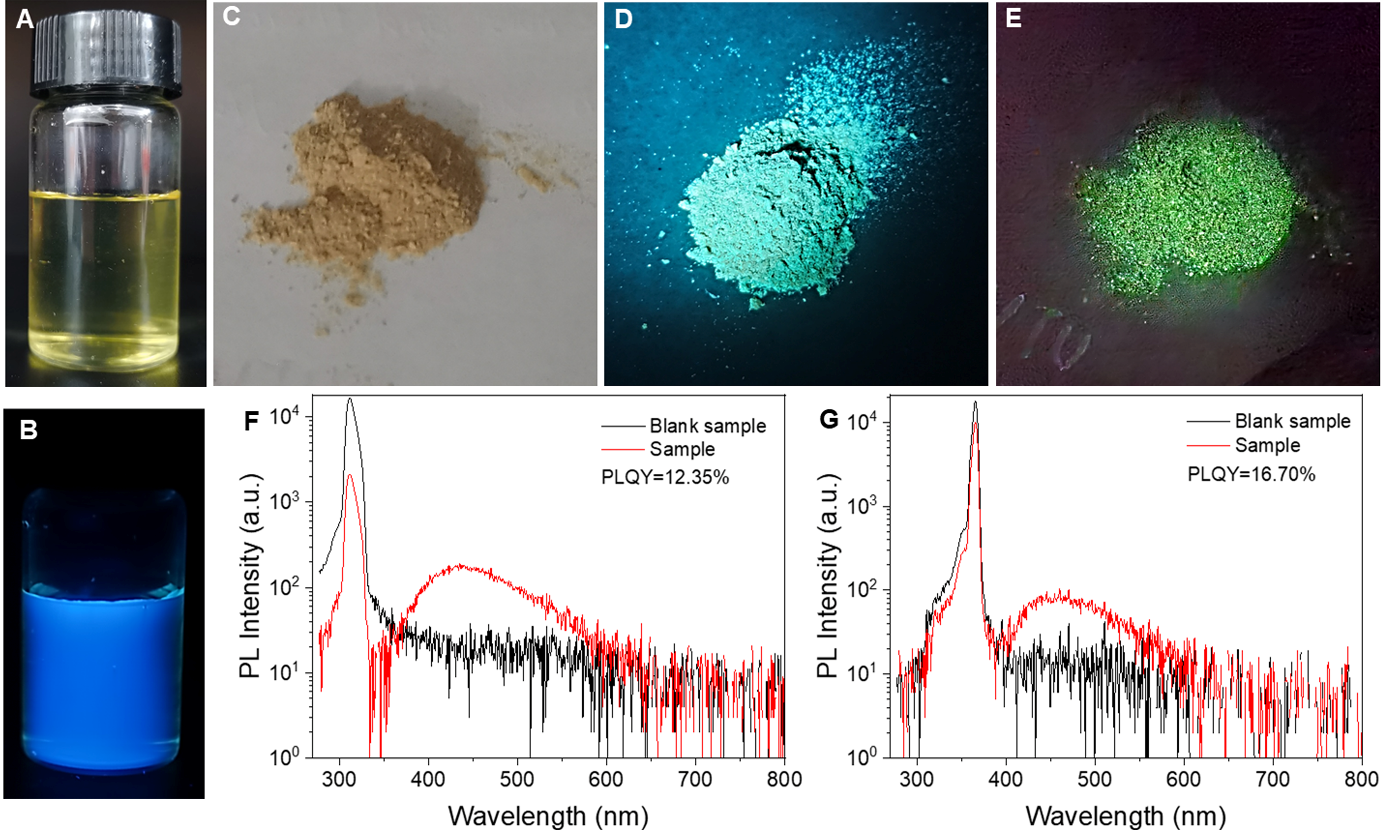


**Figure S1.** Photographs of the CDs dispersed in water under daylight (A) and 365 nm UV lamp (B), the CDs powder under daylight (C), 365 nm UV lamp (D), and the removal of UV lamp irradiation (E), respectively. (F) Excitation light profiles and PL emission spectrum of blank sample (black line) and CDs powder (red line). (G) Excitation light profiles and PL emission spectrum of blank sample (black line) and CDs aqueous-dispersion (red line).


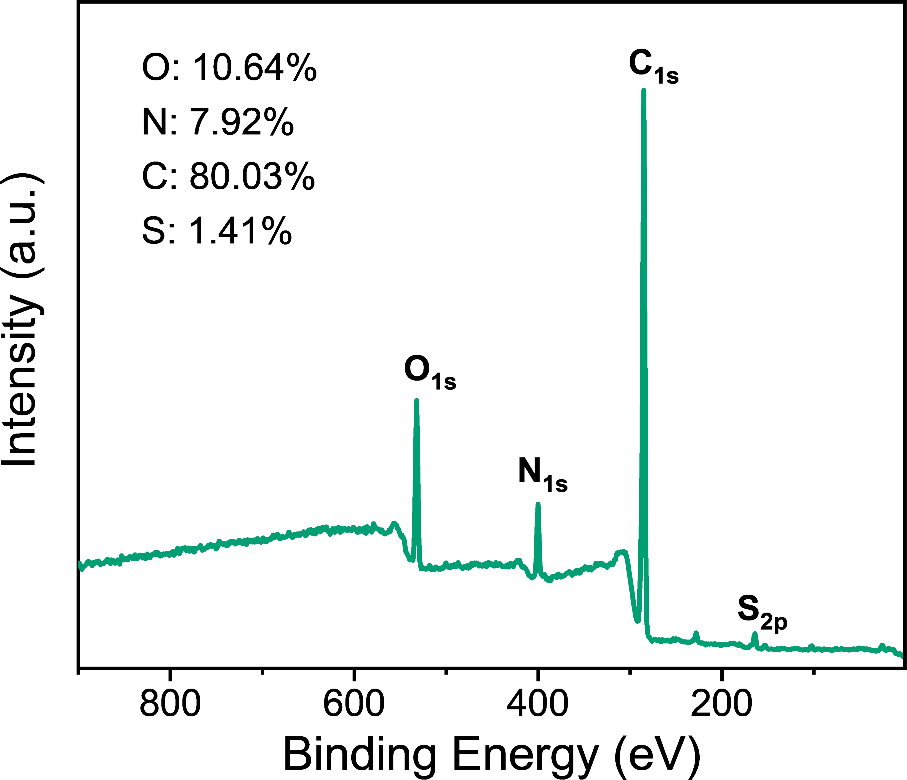


**Figure S2**. The full survey of XPS spectrum of feather meal.


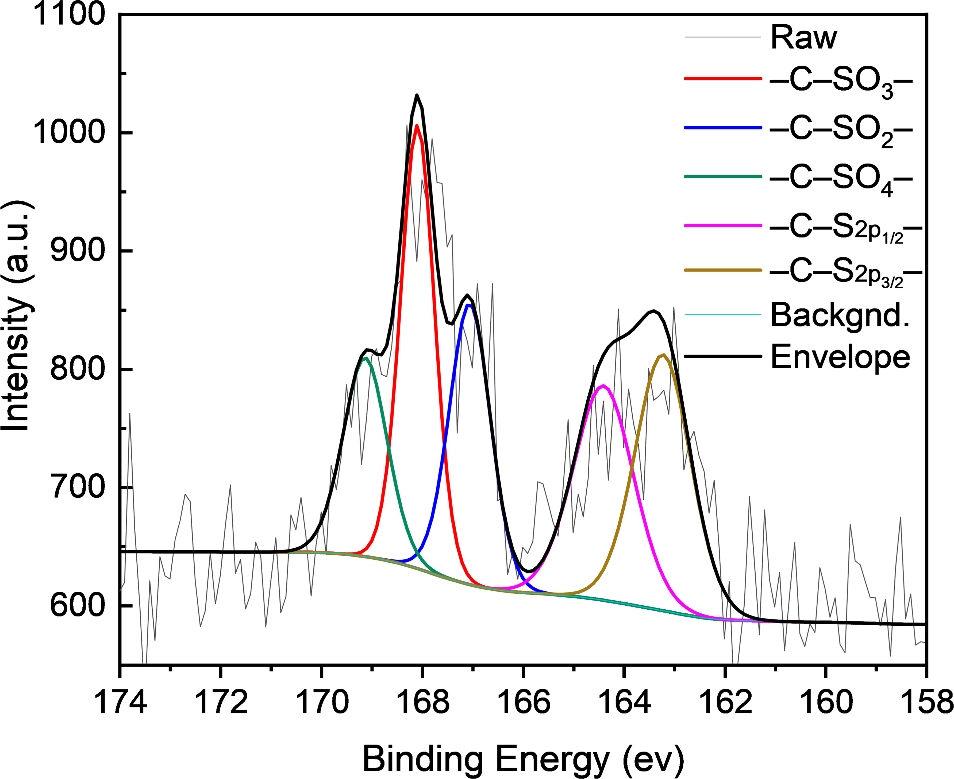


**Figure S3**. High-resolution XPS data of S 2p of the CDs.


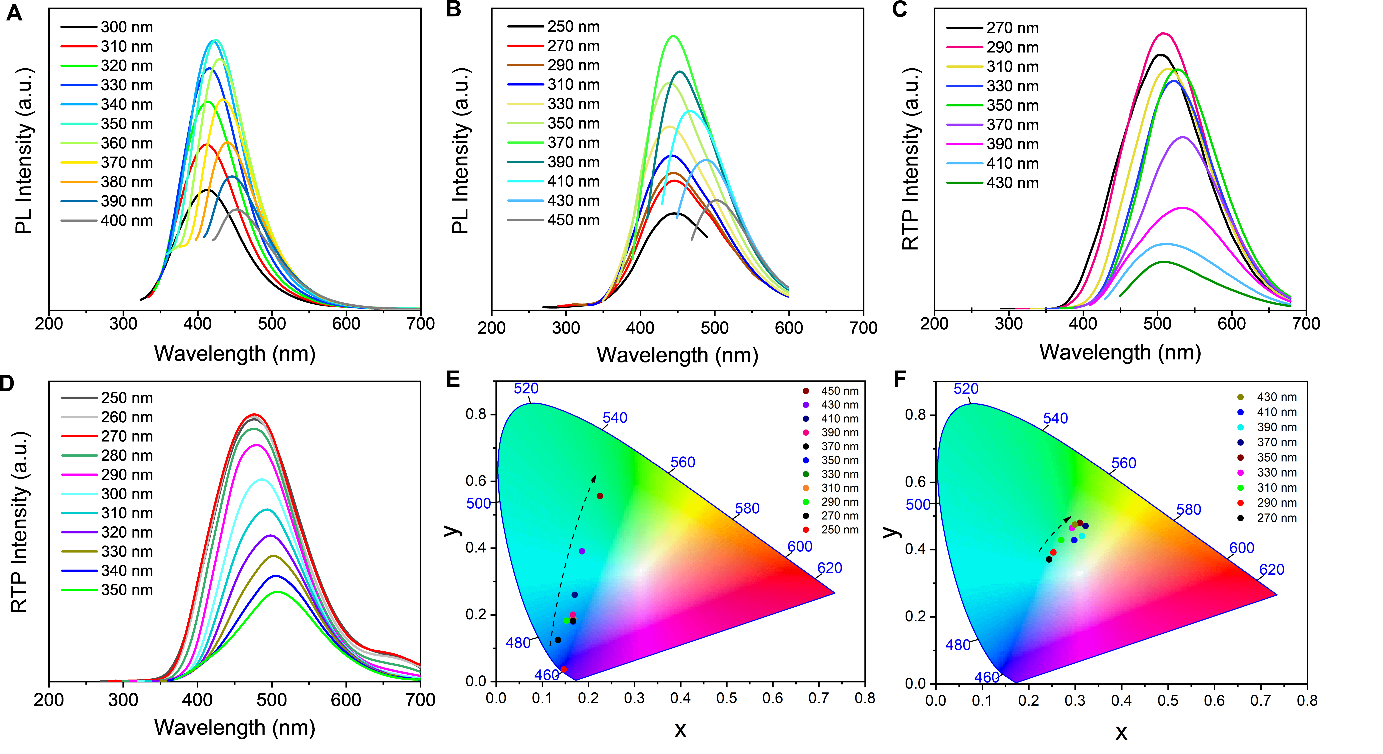


**Figure S4**. Fluorescent spectra of the water-dispersed CDs (A), and solid-state CDs (B); RTP spectra of solid-state CDs (C), and solid-circle pattern printed on cotton fabric using CPP-2 ink (D). CIE 1931 chromaticity diagram: PL emission (E) and RTP emission (F) color coordinates of the aggregated solid powder CDs under irradiations of varying wavelengths.


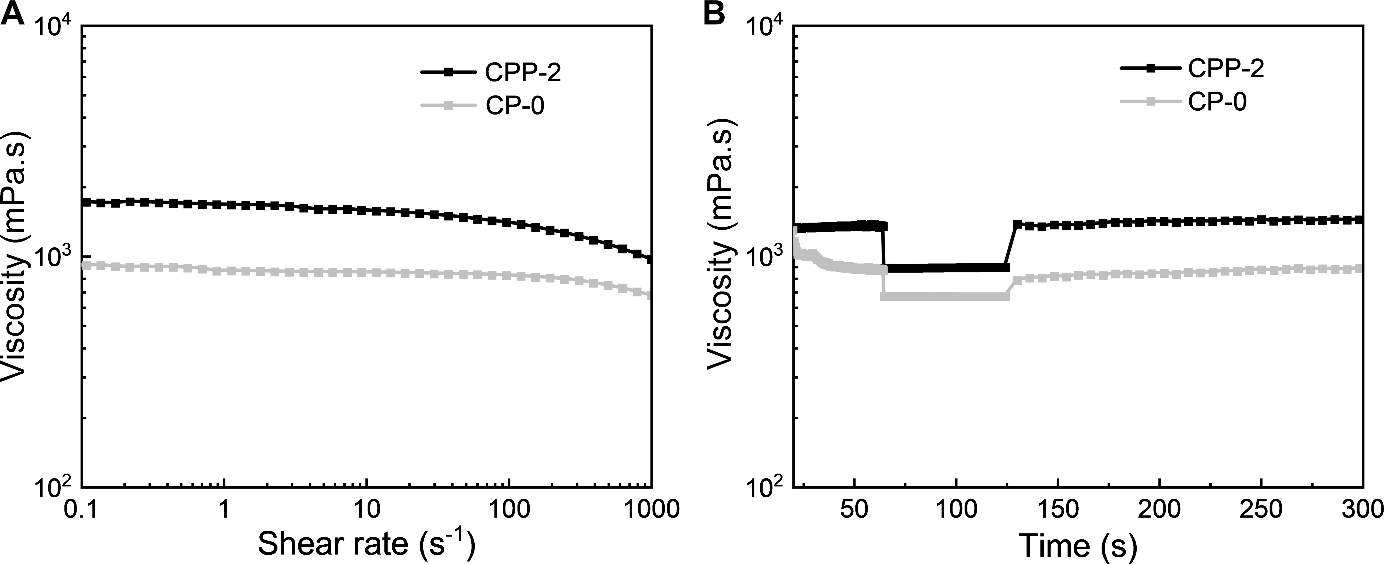


**Figure S5**. Rheological properties of the representative inks: CPP-2 (with PA added) and CP-0 (without PA added): A) the relationship between viscosity and the shear rate, B) shear viscosity versus time at three different shear rates (0.1 s^-1^ for 60 s, 1000 s^-1^ for 60 s, and 0.1 s^-1^ for 10 min). (CPP: CDs/PVA/PA)


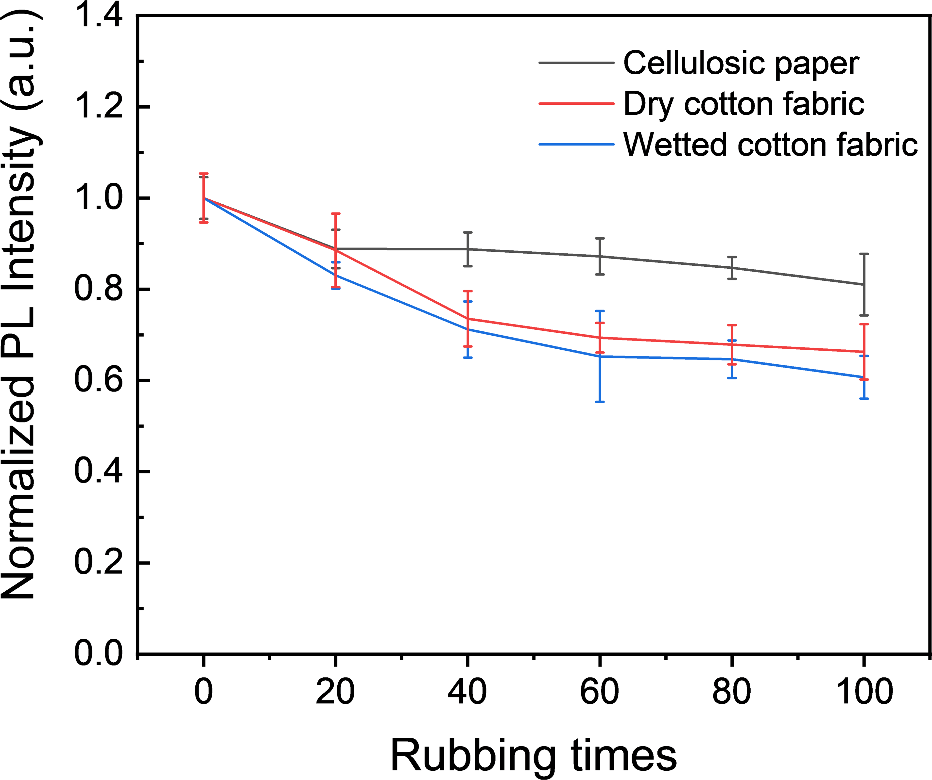


**Figure S6**. Effect of rubbing times on PL intensity of the QR code printed on cotton fabric and cellulosic paper.

**
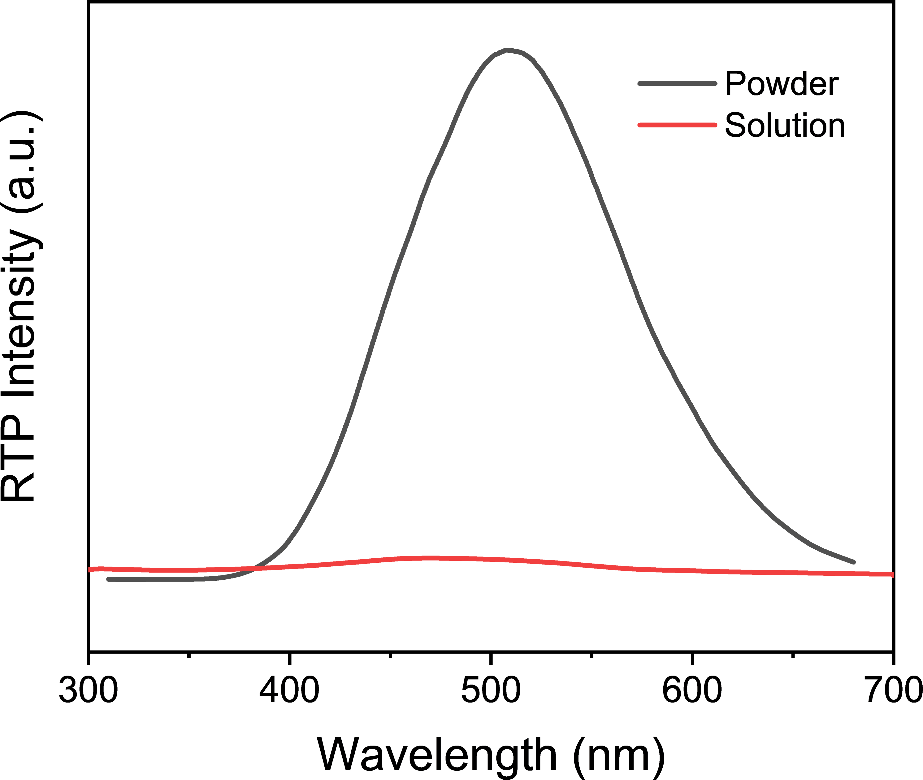
**

**Figure S7**. RTP emission spectrum of dried CDs powder (black line) and water-wetted CDs aqueous solution (red line). (RTP: Room-temperature phosphorescence)

**Table S1**. Comparison for optical properties of bio-mass CDs in this work and related reports.

| Biomass Precursors | Luminescence Modes | Fluorescence lifetime in aqueous dispersion | Solid-state fluorescence lifetime | Solid-state phosphorescence lifetime | References |
| --- | --- | --- | --- | --- | --- |
| Feather | FLUOR, RTP | 2.52 ns | 2.88 ns | 0.50 s | This work |
| Sericin | FLUOR, RTP | 5.62 ns | 4.33 ns | 13.27 ms | [1] |
| Turtle shells | FLUOR | / | 3.4 ns | / | [2] |
| Lignin  Alkali lignin | FLUOR  FLUOR | 1.30-2.94 ns  6.11 ns | /  / | /  / | [3]  [4] |
| Mulberry-Leaves | FLUOR | 6.3 ns | / | / | [5] |
| Crab shells | FLUOR | / | / | / | [6, 7] |
| Silkworm chrysalis | FLUOR | / | / | / | [8] |
| Chicken bone powder | FLUOR | / | / | / | [9] |
| Dried shrimp | FLUOR | 7.53 ns | / | / | [10] |
| Wool/pig hair | FLUOR | / | / | / | [11] |
| Overcooked barbeque meat | FLUOR | / | / | / | [12] |
| Waste fish scales | FLUOR | / | / | / | [13] |
| Hair | FLUOR | 2.83 ns | / | / | [14] |
| Silkworm excrement | FLUOR | / | / | / | [15] |
| Goose feathers | FLUOR | / | / | / | [16] |
| Chicken eggshell membrane | FLUOR | / | / | / | [17] |
| Wolfberry | FLUOR | / | / | / | [18] |
| Watermelon peel | FLUOR | 5.72 ns | / | / | [19] |
| Ginkgo leaves | FLUOR | 5.36 ns | / | / | [20] |
| Coffee powder | FLUOR | 4.64 ns | / | / | [21] |
| Natural lignocellulose | FLUOR | / | / | / | [22] |
| Exocarpium Citri Grandis | FLUOR | 2.51 ns | / | / | [23] |
| Wedelia trilobata | FLUOR | / | 4.82 ns | / | [24] |
| pakchoi | FLUOR | / | 6.8 ns | / | [25] |
| Lotus root powder | FLUOR | / | / | / | [26] |
| Pomelo peels, orange juice and strawberry juice | FLUOR | / | / | / | [27] |
| Papaya | FLUOR | / | / | / | [28] |
| Unripe peach | FLUOR | / | / | / | [29] |
| Konjac flour | FLUOR | / | / | / | [30] |
| Rose petals | FLUOR | / | / | / | [31] |
| Milk protein casein | FLUOR | / | / | / | [32] |
| Cigarette ash. | FLUOR | / | / | / | [33] |
| Neem leaves | FLUOR | / | / | / | [34] |
| Beer | FLUOR | 4.79 ns | / | / | [35] |
| Fried food waste residue. | FLUOR | / | / | / | [36] |
| Chitosan | FLUOR  FLUOR | /  6.71 ns | /  / | /  / | [37]  [38] |
| Banana peels | FLUOR | / | / | / | [39] |
| Shark cartilage | FLUOR | / | / | / | [40] |
| Milk | FLUOR | / | / | / | [41] |
| Black tea | FLUOR | / | / | / | [42] |
| Zucchini waste | FLUOR | / | / | / | [43] |

FLUOR: Fluorescence, RTP: Room-temperature phosphorescence

**Table S2.** Recovery rates of CPP inks during printing simulation.

| Sample | CPP-1 | CPP-2 | CP-0 | CPP-3 | CPP-4 |
| --- | --- | --- | --- | --- | --- |
| Viscosity at 0.1 s^-1^ for 60 s (mPa·s) | 273 | 1381 | 870 | 9424 | 64235 |
| Viscosity at 0.1 s^-1^ for 130 s (mPa·s) | 161 | 1381 | 789 | 7698 | 31005 |
| Recovery ratio within 130 s | 59% | 100% | 91% | 82% | 48% |

CPP: CDs/PVA/PA

**References**

[1] S. Li, H. Wang, H. Lu, X. Liang, H. Wang, M. Zhang, K. Xia, Z. Yin, Y. Zhang, X. Zhang, Y. Zhang, *Small* **2021**, 17, 2103623.

[2] J. Guo, H. Li, L. Ling, G. Li, R. Cheng, X. Lu, A.-Q. Xie, Q. Li, C.-F. Wang, S. Chen, *ACS Sustainable Chem. Eng.* **2020**, 8, 1566.

[3] L. Zhu, D. Shen, Q. Wang, K. H. Luo, *ACS Appl. Mater. Interfaces* **2021**, 13, 56465.

[4] T. Zhang, J. P. Zhou, H. M. Li, J. L. Ma, X. Wang, H. Q. Shi, M. H. Niu, Y. S. Liu, F. S. Zhang, Y. Z. Guo, *Green Chem.* **2023**, 25, 1406.

[5] V. L. John, A. R. Nayana, T. R. Keerthi, A. K. K. A., B. C. P. Sasidharan, V. T. P., *Macromol. Biosci.* **2023,** 2300081.

[6] K. Dehvari, K. Y. Liu, P.-J. Tseng, G. Gedda, W. M. Girma, J.-Y. Chang, *J. Taiwan Inst. Chem. Eng.* **2019**, 95, 495

[7] D. Elango, J. S. Packialakshmi, V. Manikandan, P. Jayanthi, *Materials Letters* **2022**, 313, 131822.

[8] J. Feng, W.-J. Wang, X. Hai, Y.-L. Yu, J.-H. Wang, *Journal of Materials Chemistry B* **2016**, 4, 387.

[9] W. S. B. Dwandaru, E. K. Sari, *Journal of Physical Science* **2020**, 31, 113.

[10] S. L. D'Souza, B. Deshmukh, J. R. Bhamore, K. A. Rawat, N. Lenka, S. K. Kailasa, *RSC Adv.* **2016**, 6, 12169.

[11] S. Wang, H. Niu, S. He, Y. Cai, *RSC Adv.* **2016**, 6, 107717.

[12] J. Wang, S. Sahu, S. K. Sonkar, K. N. Tackett II, K. W. Sun, Y. Liu, H. Maimaiti, P. Anilkumar, Y.-P. Sun, *RSC Adv.* **2013**, 3, 15604.

[13] Q. F. Yao, D. S. Zhou, J. H. Yang, W. T. Huang, *Sustain. Chem. Pharm.* **2020**, 17, 100305.

[14] S.-S. Liu, C.-F. Wang, C.-X. Li, J. Wang, L.-H. Mao, S. Chen, *J. Mater. Chem. C* **2014**, 2, 6477.

[15] X. Lu, C. Liu, Z. Wang, J. Yang, M. Xu, J. Dong, P. Wang, J. Gu, F. Cao, *Nanomaterials* **2018**, 8, 443.

[16] R. Liu, J. Zhang, M. Gao, Z. Li, J. Chen, D. Wu, P. Liu, *RSC Adv.* **2015**, 5, 4428.

[17] H. Zhang, S. Wu, Z. Xing, H.-B. Wang, *Analyst.* **2021**, 146, 7250.

[18] L. Gu, J. Zhang, G. Yang, Y. Tang, X. Zhang, X. Huang, W. Zhai, E. K. Fodjo, C. Kong, *Food Chem.* **2022**, 376, 131898.

[19] J. Zhou, Z. Sheng, H. Han, M. Zou, C. Li, *Mater. Lett.* **2012**, 66, 222.

[20] X. Jiang, D. Qin, G. Mo, J. Feng, C. Yu, W. Mo, B. Deng, *J. Pharmaceut. Biomed.* **2019**, 164, 514.

[21] C. Jiang, H. Wu, X. Song, X. Ma, J. Wang, M. Tan, *Talanta* **2014**, 127, 68.

[22] X. Tao, M. Liao, F. Wu, Y. Jiang, J. Sun, S. Shi, *Chem. Eng. J.* **2022**, 443, 136442.

[23] Q. Cen, F. Fu, H. Xu, L. Luo, F. Huang, J. Xiang, W. Li, X. Pan, H. Zhang, M. Zheng, Y. Zheng, Q. Li, B. Lei, *J. Mater. Chem. B* **2022**, 10, 6433.

[24] C. Liang, X. Xie, D. Zhang, J. Feng, S. Lu, Q. Shi, *J. Mater. Chem. B* **2021**, 9, 5670.

[25] Y. Cui, R. Liu, F. Ye, S. Zhao, *Nanoscale* **2019**, 11, 9270.

[26] L. Liu, X. Yu, Z. Yi, F. Chi, H. Wang, Y. Yuan, D. Li, K. Xu, X. Zhang, *Nanoscale* **2019**, 11, 15083.

[27] W. B. Lu, X. Y. Qin, S. Liu, G. H. Chang, Y. W. Zhang, Y. L. Luo, A. M. Asiri, A. O. Al-Youbi, X. P. Sun, *Anal. Chem.* **2012**, 84, 5351.

[28] N. Wang, Y. T. Wang, T. T. Guo, T. Yang, M. L. Chen, J. H. Wang, *Biosens. Bioelectron.* **2016**, 85, 68.

[29] R. Atchudan, T. Edison, Y. R. Lee, *J. Colloid Interface Sci.* **2016**, 482, 8.

[30] X. Y. Teng, C. G. Ma, C. J. Ge, M. Q. Yan, J. X. Yang, Y. Zhang, P. C. Morais, H. Bi, *J. Mat. Chem. B* **2014**, 2, 4631.

[31] Y. J. Feng, D. Zhong, H. Miao, X. M. Yang, *Talanta* **2015**, 140, 128.

[32] S. K. Bajpai, A. D'Souza, B. Suhail, *Int. Nano Lett.* **2019**, 9, 203.

[33] H. Y. Huang, Y. Cui, M. Y. Liu, J. Y. Chen, Q. Wan, Y. Q. Wen, F. J. Deng, N. G. Zhou, X. Y. Zhang, Y. Wei, *J. Colloid Interface Sci.* **2018**, 532, 767.

[34] A. Suryawanshi, M. Biswal, D. Mhamane, R. Gokhale, S. Patil, D. Guin, S. Ogale, *Nanoscale* **2014**, 6, 11664.

[35] Z. Y. Wang, H. Liao, H. Wu, B. B. Wang, H. D. Zhao, M. Q. Tan, *Anal. Methods* **2015**, 7, 8911.

[36] E. Dhandapani, N. Duraisamy, R. Mohan Raj, *Materials Today: Proceedings* **2022**, 51, 1696.

[37] Y. H. Yang, J. H. Cui, M. T. Zheng, C. F. Hu, S. Z. Tan, Y. Xiao, Q. Yang, Y. L. Liu, *Chem. Commun.* **2012**, 48, 380.

[38] Y. Y. Ni, P. Y. Zhou, Q. W. Jiang, Q. Zhang, X. Y. Huang, Y. Jing, *Dyes Pigment.* **2022**, 197, 9.

[39] R. Atchudan, T. N. Jebakumar Immanuel Edison, M. Shanmugam, S. Perumal, T. Somanathan, Y. R. Lee, *Physica E Low Dimens. Syst. Nanostruct.* **2021**, 126, 114417.

[40] K. W. Kim, T. Y. Choi, Y. M. Kwon, J. Y. H. Kim, *Electron. J. Biotechnol.* **2020**, 47, 36.

[41] Y. F. Yuan, B. Guo, L. Y. Hao, N. Liu, Y. F. Lin, W. S. Guo, X. G. Li, B. Gu, *Colloids Surf. B* **2017**, 159, 349.

[42] S. Bayda, M. Hadla, S. Palazzolo, V. Kumar, I. Caligiuri, E. Ambrosi, E. Pontoglio, M. Agostini, T. Tuccinardi, A. Benedetti, P. Riello, V. Canzonieri, G. Corona, G. Toffoli, F. Rizzolio, *J. Control. Release* **2017**, 248, 144.

[43] V. C. Hoang, V. G. Gomes, *Mater. Today Energy* **2019**, 12, 198.
